# Extra-Acral Minute Synovial Sarcoma: A Case Report With Literature Review

**DOI:** 10.7759/cureus.67505

**Published:** 2024-08-22

**Authors:** John Grove, Rana Naous

**Affiliations:** 1 Pathology and Laboratory Medicine, University of Pittsburgh Medical Center, Pittsburgh, USA; 2 Pathology, University of Pittsburgh Medical Center, Pittsburgh, USA

**Keywords:** acral, extra-acral, sarcoma, synovial, minute

## Abstract

Synovial sarcoma is a malignant soft tissue tumor of uncertain differentiation. It is typically seen in the deep soft tissue of the extremities; however, it has been reported to occur anywhere in the body. Synovial sarcoma by histomorphology has multiple subtypes, including monophasic spindle cell, biphasic and poorly differentiated subtypes. Synovial sarcomas measuring less than one centimeter in diameter are termed “minute” synovial sarcomas. “Minute” synovial sarcomas have only been reported so far in the acral region of the hands and feet. They are extremely rare and can often be misinterpreted as benign neoplasms. Herein, we report the findings in a 30-year-old female presenting with a palpable mass within the deep subcutaneous tissue along the anterior aspect of her right rectus abdominis muscle. The mass was excised and measured 0.6 cm in greatest dimension with histomorphology findings, immunohistochemical and molecular workup confirming the diagnosis of “minute” synovial sarcoma. Our findings represent the first documented case of a “minute” synovial sarcoma occurring at an extra-acral site. With such unique finding not yet reported in the literature, this case highlights the importance of considering synovial sarcoma in the differential diagnosis of subcutaneous abdominal masses.

## Introduction

Synovial sarcomas (SS) are soft tissue sarcomas that comprise roughly 5-10% of all soft tissue neoplasms [1]. Synovial sarcomas develop secondary to a unique chromosomal translocation t(X;18)(p11;q11) involving SS18, SSX1, SSX2 or SSX4 genes resulting in a characteristic SS18-SSX fusion [2,3]. Histologically, synovial sarcomas have a monomorphic blue spindle cell appearance and can appear as either monophasic, biphasic, or poorly differentiated, with the monophasic subtype being the most common [4]. SS typically affect males and females equally and are most commonly seen in young adults with a median age at diagnosis of 30 years old [5]. Most synovial sarcomas measure more than 3 cm in size; however, when a synovial sarcoma is less than one centimeter in greatest dimension, it is defined as a “minute” synovial sarcoma. Minute SS are almost exclusively seen in the hands and feet [6]. Herein, we report the first extra-acral documented case of a “minute” synovial sarcoma located outside the extremities in a 30-year-old female presenting with a palpable mass on her lower abdomen.

## Case presentation

**Figure 1:**
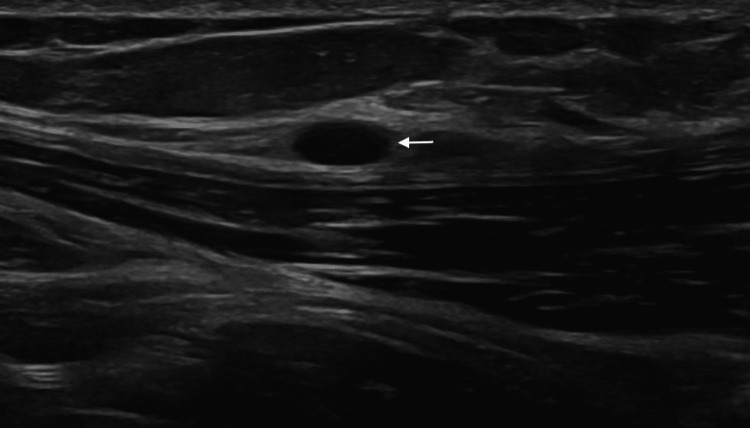
Ultrasound imaging Ultrasound imaging showing a small heterogeneously hypoechoic mass in the subcutaneous fat; arrow at mass.

**Figure 2:**
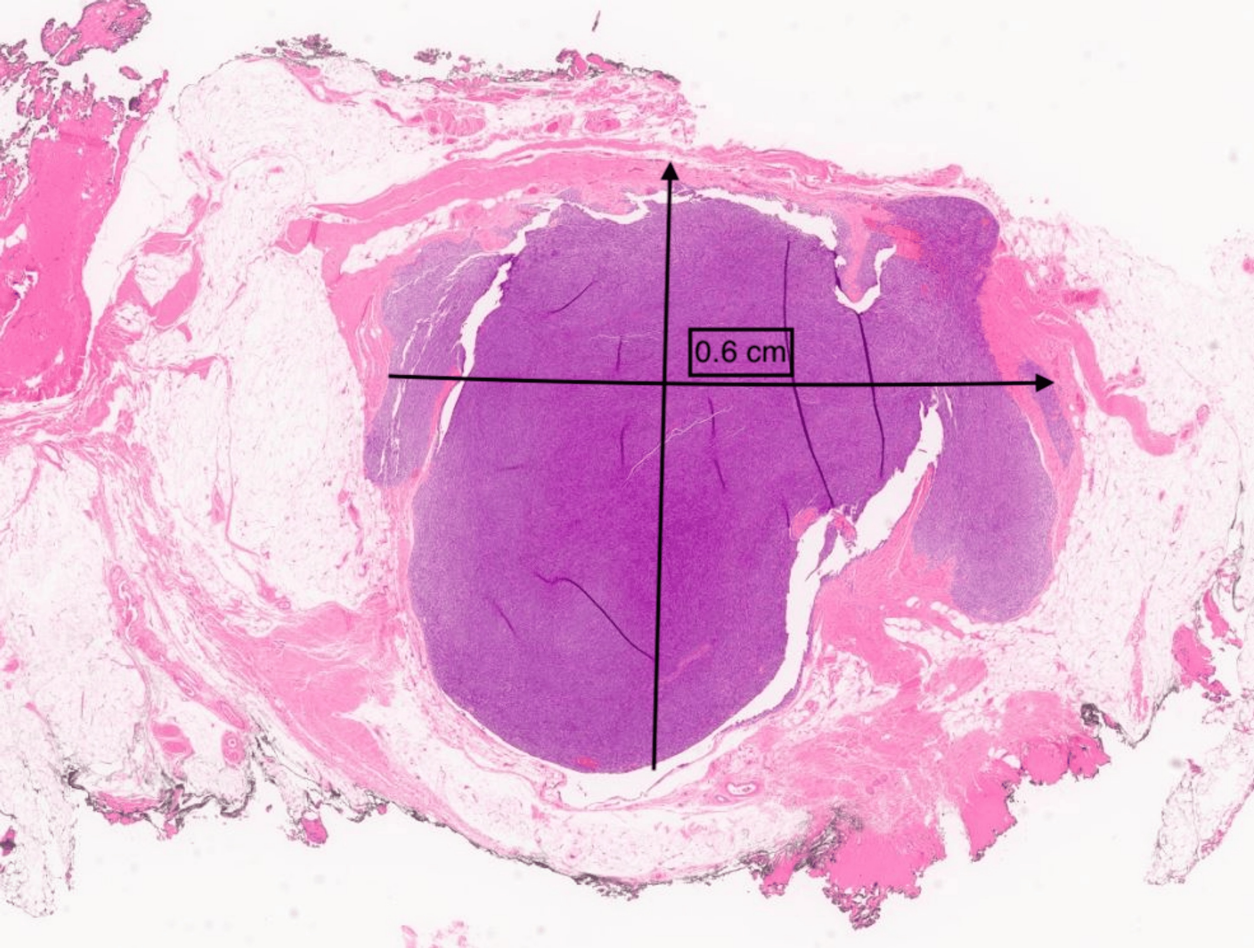
Minute synovial sarcoma (at low power magnification) Minute synovial sarcoma involving abdominal subcutaneous tissue and measuring 0.6 cm in maximum dimension (hematoxylin and eosin, 4x).

**Figure 3:**
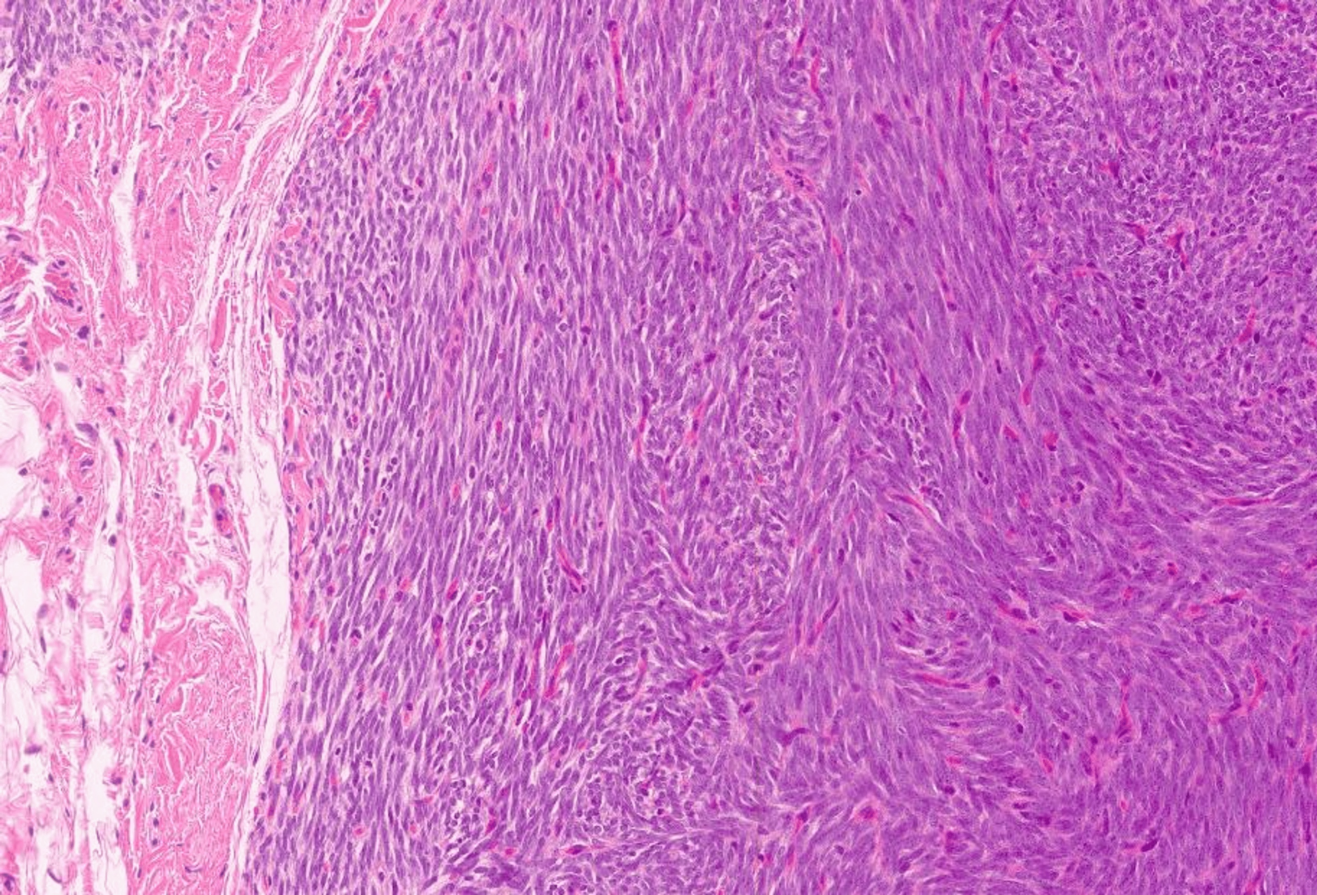
Minute synovial sarcoma (at high power magnification) Minute synovial sarcoma comprised of monomorphic hyperchromatic spindle cells with vesicular chromatin and minimal cytoplasm arranged in compact fascicles (hematoxylin and eosin, 20x).

**Figure 4:**
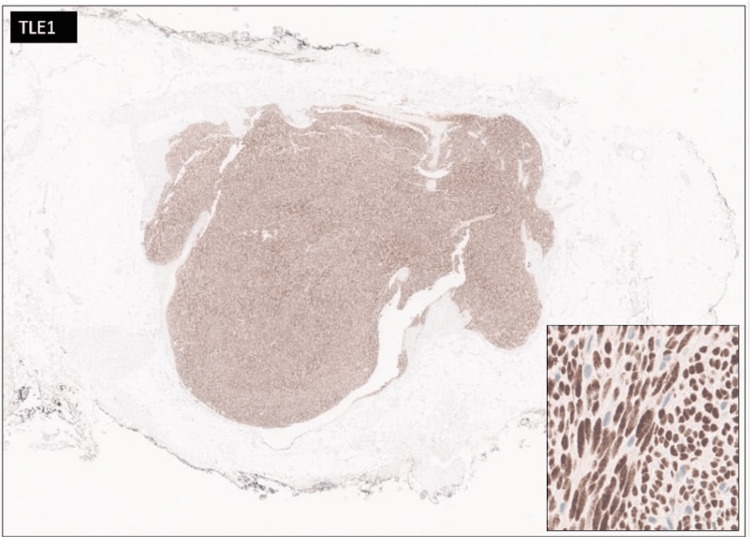
TLE1 immunostain Diffuse positive immunohistochemical staining for TLE1 in the tumor cells (TLE1 immunostain, 4x). Inset at higher power magnification highlighting the positive nuclear staining within the tumor cells (TLE1 immunostain, 20x).

**Figure 5:**
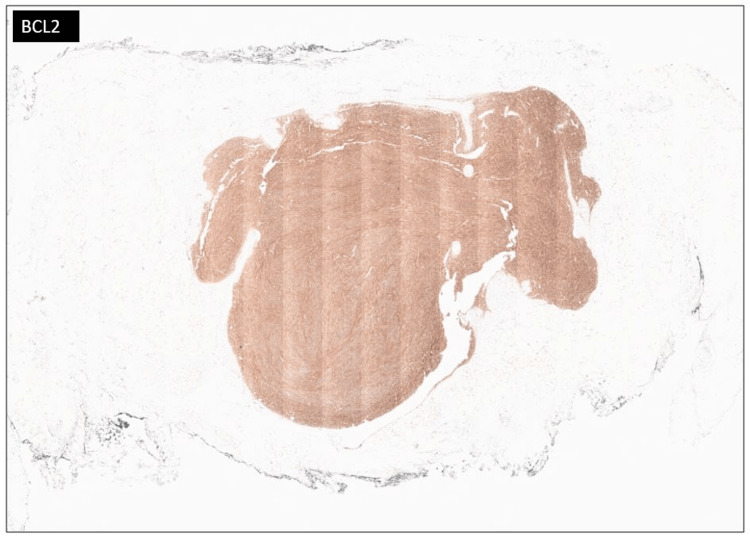
BCL2 immunostain Diffuse positive immunohistochemical staining for BCL2 in the tumor cells (BCL2 immunostain, 4x).

**Figure 6:**
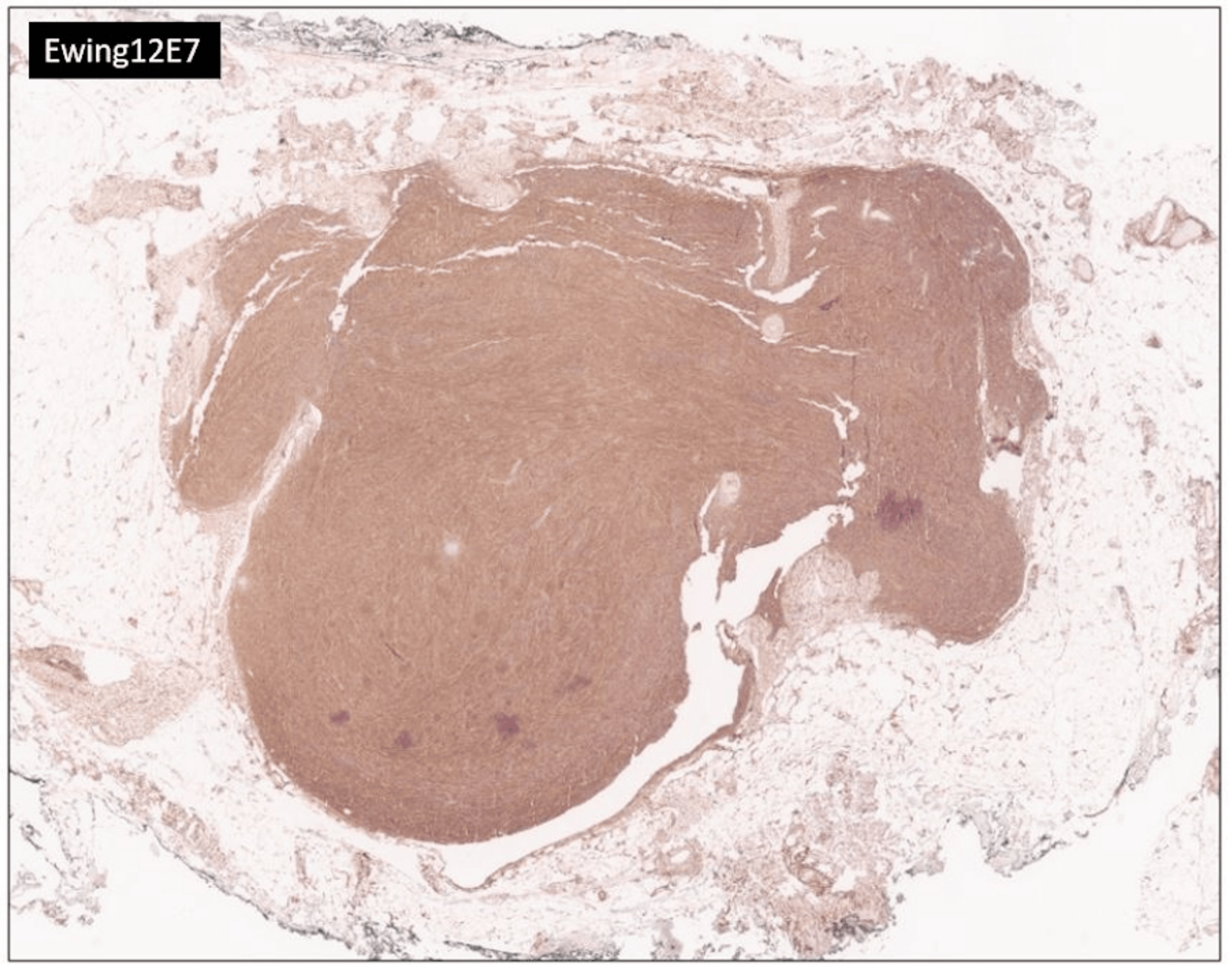
Ewing 12E7 immunostain Diffuse positive immunohistochemical staining for Ewing 12E7 in the tumor cells (Ewing 12E7 immunostain, 4x).

**Figure 7:**
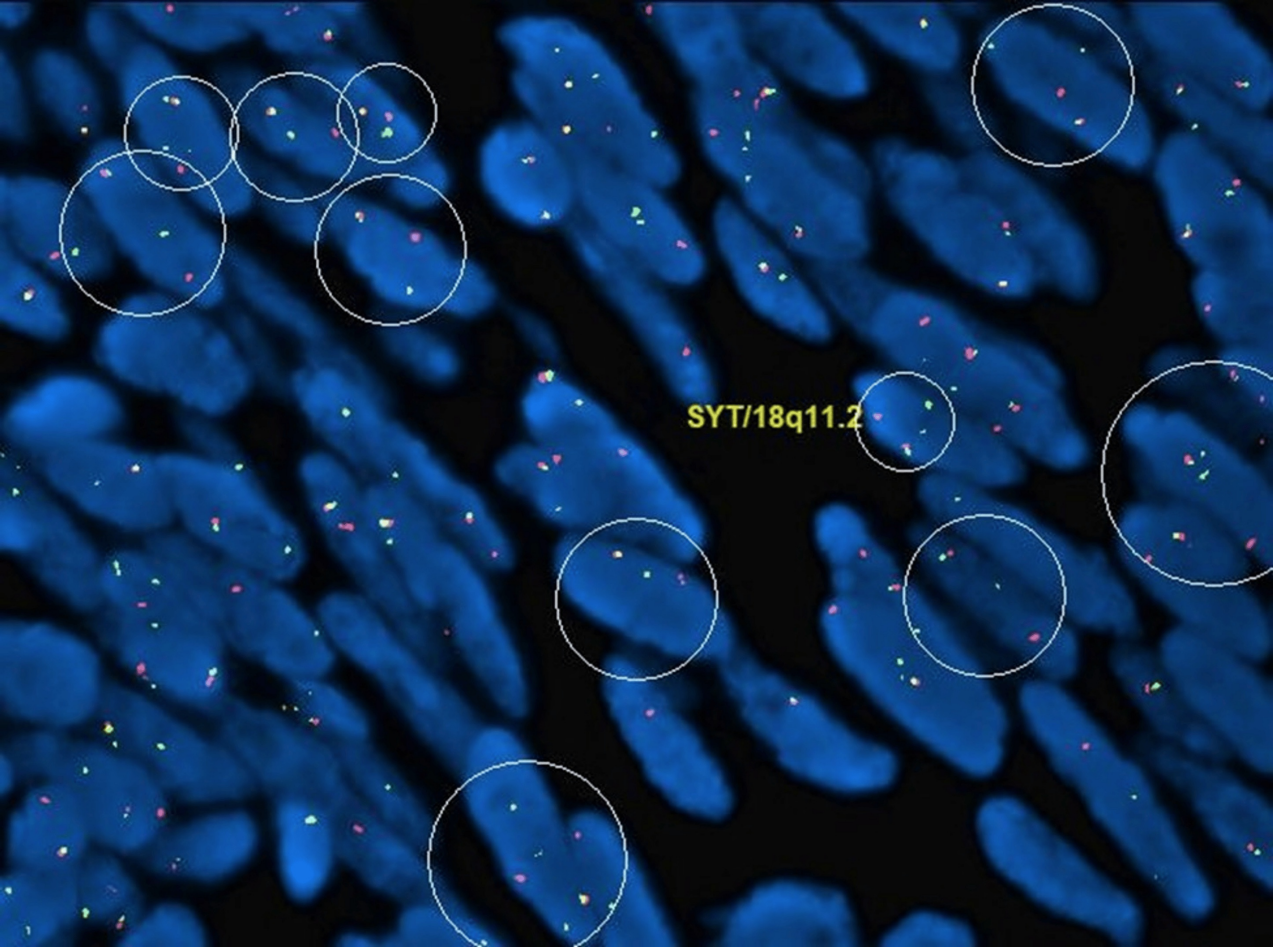
SS18 (SYT) FISH analysis SS18 (SYT) FISH analysis was positive for translocation. A translocated cell contains a fused orange [SS18 SpectrumOrange -telomeric] and green signal [SS18 SpectrumGreen -centromeric] probes, one separate green signal, and one separate orange signal.

A 30-year-old female presented with a chief complaint of a right lower quadrant abdominal mass. The patient first noticed the mass three years ago. The mass was tender to palpation. The patient reported the tenderness was perimenstrual and worsened by flexion of the abdominal muscles. A complete blood count (CBC), comprehensive metabolic panel, lipid panel and hepatic function panel were performed and were non-contributory. A CT scan of the abdomen performed at an outside institution (images not available) noted a 7 mm ovoid enhancing lesion within the deep subcutaneous tissues along the anterior aspect of the right rectus abdominis muscle. An ultrasound done in-house showed a small heterogeneously hypoechoic mass with indistinct margins and internal blood flow in the deep subcutaneous fat, potentially infiltrating the rectus abdominis fascia (Figure 1). As part of the therapeutic management, the patient underwent an uncomplicated resection of the abdominal mass which was subsequently sent to pathology. The specimen received was labeled “Right lower quadrant subcutaneous mass" and consisted of a 2.0 x 1.7 x 0.8 cm irregular portion of tan-white rubbery fibroadipose tissue. The specimen was serially sectioned to reveal tan, white to tan-pink cut surfaces with no gross evidence of masses or lesions. Microscopically, an incidental tumor mass was identified and measured 0.6 cm in greatest dimension (Figure 2). The tumor was composed of monomorphic, hyperchromatic spindle cells with vesicular chromatin and minimal cytoplasm arranged in compact fascicles (Figure 3). The tumor showed mild mitotic activity with up to 6 mitoses per 10 high power field and no evidence of necrosis. Immunohistochemical stains were diffusely positive in the tumor cells for TLE1 (Figure 4), BCL2 (Figure 5), and Ewing 12E7 (Figure 6), and negative for STAT6, ERG and CD34. Fluorescence in situ hybridization (FISH) for the SS18 (SYT) gene rearrangement was positive (Figure 7); thus, confirming the diagnosis of “minute” synovial sarcoma. The patient subsequently underwent staging imaging which came back negative for metastatic disease and is currently planned for tumor bed-wide resection.

## Discussion

Synovial sarcomas are malignant soft tissue tumors, the majority of which are found in the extremities with other common locations being the trunk, and the head and neck regions among others [4]. Synovial sarcomas are regarded as tumors of uncertain differentiation; however, it has been hypothesized that a human multipotent mesenchymal stem cell may be their cell of origin [7].

The underlying pathophysiology that drives the progression of synovial sarcomas is the t(X;18)(p11;q11) chromosomal translocation which affects multiple oncogenic pathways including the Wnt pathway and the SWI/SNF chromatin remodeling complex [8]. SS18 gene is present on chromosome 18 and encodes a subunit of the SWI/SNF (BAF) complex, which has a role in chromatin remodeling. Fusions involving SS18 and the SSX gene family (SSX1, SSX2, SSX4) replace SS18 C-terminus with a portion of the SSX C-terminus. This subsequently displaces wild-type SS18 and alters the SWI/SNF function to promote proliferation [9-10]. The most common 3' partners of the SS18 gene are SSX1 and SSX2. Tumors with SSX2 fusions commonly show monophasic spindle cell morphology, and up to 30-40% SSX1 fusions show biphasic histology. The type of SSX fusion gene does not seem to affect prognosis [11-14].

Synovial sarcomas, albeit being commonly associated with the extremities, rarely occur in acral sites with a reported frequency of 4-8.5% of all synovial sarcomas [15-17].

Minute synovial sarcomas, those that are less than one centimeter in diameter, are almost always found in the acral regions of the extremities. Michal et al. [6] evaluated 21 minute synovial sarcomas that are less than 1 cm in diameter from acral sites of the hands and feet. In their cohort, one-third of the tumors were biphasic and two-thirds were monophasic spindle cell variants. All their patients with complete follow-up had no evidence of disease after treatment. Subsequently, the authors concluded that minute synovial sarcomas of hands and feet have a clinically favorable outcome if completely excised with some evidence suggesting conservative surgical management.

With a large majority of synovial sarcomas arising in the extremities, the diagnosis can be challenging when the location is outside of a typical area. Immunohistochemistry and molecular testing play a crucial role in confirming the diagnosis, typically showing immunopositivity for cytokeratin, EMA, BCL2, and CD99 immunostains, as well as detection of the characteristic SS18 gene fusion via FISH. In our case, the tumor measured <1 cm and occurred in an unusual extra-acral site within the subcutaneous tissue of the abdomen. Our immunohistochemical panel and FISH SS18 studies confirmed the diagnosis of an extra-acral “minute” synovial sarcoma, a unique and rarely occurring entity.

Synovial sarcoma has three main morphologic subtypes: monophasic, biphasic and poorly differentiated. The monophasic subtype is comprised of variably cellular and dense sheets of uniform spindle cells with little cytoplasm in a background of minimal to increased collagenous stroma and variable mitotic rate. The biphasic synovial sarcoma subtype harbors a similar morphology to the monophasic subtype but with an additional focal to extensive epithelial component arranged in nests, cords, ducts or glands. The poorly differentiated subtype is characterized by high-grade features, increased mitosis, prominent nucleoli, hypercellularity, occasional necrosis and/or round cell morphology reminiscent of “Ewing sarcoma” [4]. Our case belonged to the monophasic spindle cell subtype; however, as this is the first reported case of an extra-acral minute synovial sarcoma, it remains to be determined whether this subtype is commonly associated with “minute” synovial sarcomas at extra-acral sites or not.

Synovial sarcomas in general have an estimated range of overall five-year survival rate ranging between 27-85% [18, 19]. Surgical excision with wide margins remains the cornerstone of treatment for localized synovial sarcoma. The role of adjuvant therapies such as radiotherapy or chemotherapy in such cases is controversial and often tailored based on poor prognostic risk factors such as tumor size (tumors larger than 4 or 5 cm), grade (poorly differentiated histology), advanced age at diagnosis, central location and margin status [20]. Recent development of immunotherapy and targeted therapy may prove to be beneficial to patients with advanced disease [5]. Long-term prognosis is usually favorable if the neoplasm is detected early, and complete surgical resection is achieved.

## Conclusions

Herein, we report the first documented case of a “minute” synovial sarcoma occurring at an extra-acral site, in the abdominal subcutaneous tissue. “Minute” synovial sarcomas are rare pathologic entities with few cases reported in the literature. To our knowledge, all “minute” synovial sarcomas thus far have been reported to occur in acral sites of the hands or feet. Our patient represents the first documented case to occur outside such locations. With such a unique finding not yet reported in the literature, this case highlights the importance of considering synovial sarcoma in the differential diagnosis of subcutaneous abdominal masses. In addition, early recognition and prompt treatment of “minute” synovial sarcomas can lead to favorable outcomes, thus highlighting the need for awareness among pathologists regarding this rare entity.

## References

[1] Gazendam AM, Popovic S, Munir S, Parasu N, Wilson D, Ghert M (2021). Synovial sarcoma: a clinical review. Curr Oncol.

[2] Baranov E, McBride MJ, Bellizzi AM, Ligon AH, Fletcher CD, Kadoch C, Hornick JL (2020). A novel SS18-SSX fusion-specific antibody for the diagnosis of synovial sarcoma. Am J Surg Pathol.

[3] Kawai A, Woodruff J, Healey JH, Brennan MF, Antonescu CR, Ladanyi M (1998). SYT-SSX gene fusion as a determinant of morphology and prognosis in synovial sarcoma. N Engl J Med.

[4] Thway K, Fisher C (2014). Synovial sarcoma: defining features and diagnostic evolution. Ann Diagn Pathol.

[5] Blay JY, von Mehren M, Jones RL (2023). Synovial sarcoma: characteristics, challenges, and evolving therapeutic strategies. ESMO Open.

[6] Michal M, Fanburg-Smith JC, Lasota J, Fetsch JF, Lichy J, Miettinen M (2006). Minute synovial sarcomas of the hands and feet: a clinicopathologic study of 21 tumors less than 1 cm. Am J Surg Pathol.

[7] Naka N, Takenaka S, Araki N (2010). Synovial sarcoma is a stem cell malignancy. Stem Cells.

[8] Nielsen TO, Poulin NM, Ladanyi M (2015). Synovial sarcoma: recent discoveries as a roadmap to new avenues for therapy. Cancer Discov.

[9] dos Santos NR, de Bruijn DR, van Kessel AG (2001). Molecular mechanisms underlying human synovial sarcoma development. Genes Chromosomes Cancer.

[10] Kadoch C, Crabtree GR (2013). Reversible disruption of mSWI/SNF (BAF) complexes by the SS18-SSX oncogenic fusion in synovial sarcoma. Cell.

[11] Kubo T, Shimose S, Fujimori J, Furuta T, Ochi M (2015). Prognostic value of SS18-SSX fusion type in synovial sarcoma; systematic review and meta-analysis. SpringerPlus.

[12] Antonescu CR, Kawai A, Leung DH, Lonardo F, Woodruff JM, Healey JH, Ladanyi M (2000). Strong association of SYT-SSX fusion type and morphologic epithelial differentiation in synovial sarcoma. Diagn Mol Pathol.

[13] Jones KB, Barrott JJ, Xie M (2016). The impact of chromosomal translocation locus and fusion oncogene coding sequence in synovial sarcomagenesis. Oncogene.

[14] Amary MF, Berisha F, Bernardi Fdel C (2007). Detection of SS18-SSX fusion transcripts in formalin-fixed paraffin-embedded neoplasms: analysis of conventional RT-PCR, qRT-PCR and dual color FISH as diagnostic tools for synovial sarcoma. Mod Pathol.

[15] Outani H, Hamada K, Oshima K (2014). Clinical outcomes for patients with synovial sarcoma of the hand. SpringerPlus.

[16] Dreyfuss UY, Boome RS, Kranold DH (1986). Synovial sarcoma of the hand—a literature study. J Hand Surg Br.

[17] Kransdorf MJ (1995). Malignant soft-tissue tumors in a large referral population: distribution of diagnoses by age, sex, and location. AJR Am J Roentgenol.

[18] Casal D, Ribeiro AI, Mafra M, Azeda C, Mavioso C, Mendes MM, Mouzinho MM (2012). A 63-year-old woman presenting with a synovial sarcoma of the hand: a case report. J Med Case Rep.

[19] Lindberg MR (2016). Diagnostic Pathology: Soft Tissue Tumors.

[20] El Beaino M, Araujo DM, Lazar AJ, Lin PP (2017). Synovial sarcoma: advances in diagnosis and treatment identification of new biologic targets to improve multimodal therapy. Ann Surg Oncol.

